# Livestock integration into soybean systems improves long-term system stability and profits without compromising crop yields

**DOI:** 10.1038/s41598-021-81270-z

**Published:** 2021-01-18

**Authors:** Pedro Arthur de Albuquerque Nunes, Emilio Andrés Laca, Paulo César de Faccio Carvalho, Meng Li, William de Souza Filho, Taise Robinson Kunrath, Amanda Posselt Martins, Amélie Gaudin

**Affiliations:** 1 Department of Forage Plants and Agrometeorology, Federal University of Rio Grande do Sul, Porto Alegre, RS 91540-000 Brazil; 2 Department of Plant Sciences, University of California - Davis, Davis, CA 95616 USA; 3 Department of Soil Science, Federal University of Rio Grande do Sul, Porto Alegre, RS 91540-000 Brazil

**Keywords:** Agroecology, Biodiversity, Ecosystem services, Grassland ecology

## Abstract

Climate models project greater weather variability over the coming decades. High yielding systems that can maintain stable crop yields under variable environmental scenarios are critical to enhance food security. However, the effect of adding a trophic level (i.e. herbivores) on the long-term stability of agricultural systems is not well understood. We used a 16-year dataset from an integrated soybean-beef cattle experiment to measure the impacts of grazing on the stability of key crop, pasture, animal and whole-system outcomes. Treatments consisted of four grazing intensities (10, 20, 30 and 40 cm sward height) on mixed black oat (*Avena strigosa*) and Italian ryegrass (*Lolium multiflorum*) pastures and an ungrazed control. Stability of both human-digestible protein production and profitability increased at moderate to light grazing intensities, while over-intensification or absence of grazing decreased system stability. Grazing did not affect subsequent soybean yields but reduced the chance of crop failure and financial loss in unfavorable years. At both lighter and heavier grazing intensities, tradeoffs occurred between the stability of herbage production and animal live weight gains. We show that ecological intensification of specialized soybean systems using livestock integration can increase system stability and profitability, but the probability of win–win outcomes depends on management.

## Introduction

Production of domestic animals and crops have been interconnected since the early days of agriculture and integrated crop-livestock systems (ICLS) remain the cornerstone of smallholding systems and global food security^1–3^. These systems are characterized by cropland grazing and usage of services provided by animals (e.g., nutrient recycling and weed control) to reduce input needs and enhance crop yields^4,5^. Yet, industrialization and intensification of farming systems has led to increasingly specialized operations and decoupling of crops and livestock in the last decades^6,7^. These highly productive specialized systems, such as monoculture cropping and feedlots, often rely heavily on external inputs and have high environmental costs, including contamination of water resources^8,9^ and greenhouse gas emissions^10,11^.

Biologically simplified agroecosystems are also more vulnerable to extreme weather events^12–15^ and projected increases in the frequency and severity of droughts and heavy rainfall events with climate change will challenge current crop production models^16–18^. In addition, world population is projected to increase by 25% and reach 9.8 billion people by 2050^19^. Given the rising demand for food, developing and adopting high yielding sustainable production systems able to maintain crop yields under different weather scenarios is critical to maintain global food security in an increasingly challenging production environment^20–22^.

Re-coupling crops and animals to form more biodiverse agroecosystems is increasingly proposed as a strategy to reconcile high levels of food production with maintenance of fundamental ecosystem services underlying sustainability^2^. ICLS are designed to harness complementarities and synergies between soil, plants and animals across various spatiotemporal scales (e.g., within farms through seasonal pasture-crop rotations^23^ or grazing of understory vegetation in perennial cropping systems^24^). In subtropical Brazil, ICLS are usually implemented in the form of an annual rotation of cash crops followed by grass cover crops grazed by beef cattle^23^. Out of 14.7 million ha currently cultivated with summer cash crops in the region (soybean, maize and rice), only 2.5 million ha are rotated with winter cash crops and 2.2 million ha with off-season maize^25^, while the rest often has winter cover crops in no-till systems which could be grazed^23^. Considering that this is the Brazilian region with the highest levels of ICLS adoption (13% of the total area cultivated with crops is integrated with livestock)^26^, there is a large unexplored potential for ICLS implementation. Implementing commercial-scale ICLS is a complex challenge^27^ and concerns over the impact of livestock on subsequent crop yields and the greater managerial intensity and knowledge demanded by ICLS have been listed as the main barriers to adoption^23,28–30^.

Various reports have reviewed the benefits and tradeoffs of crop-livestock integration for soil quality, nutrient cycling, crop and animal production and farm economic performance in a wide range of systems and regions of the world^23,28–32^. Livestock integration increases land-use efficiency and farm profitability while providing opportunities to bolster ecological mechanisms underlying resilience^33^. Income diversification can reduce risks from uncontrolled variability in climate and market fluctuations, as annual returns from crop and livestock commodities are often uncorrelated^28,34,35^. At the field scale, self-regulating processes such as greater nutrient cycling^32^, higher microbial functional diversity^36^ and improved soil structure^37^ and organic matter^38^ in grazed systems are suggested to increase systems’ biophysical buffering capacity to less optimal environmental conditions, in ways that still require better understanding^14,37^. Livestock production within ICLS also takes advantage of crop residues and grasses inedible to humans to produce high-quality food (e.g., beef and milk byproducts), thus reducing market competition for human-edible feed resources^39^. However, if we aim to use crop-livestock integration as a tool for sustainable intensification, it is imperative to assess its contribution to not only system productivity but also stability over the long-term.

Stability has multiple meanings in ecology and statistics and encompasses concepts like resistance and resilience^14,40–42^. In this study, we considered the concept of stability as related to variability and defined a stable system as one that changes least in response to environmental changes^43^. Management approaches that promote biodiversity (e.g., organic agriculture and crop rotation diversity) and conservation practices (e.g., permanent soil cover and reduced disturbance) have been shown to enhance yield stability^13,15,22,42,44–53^. Besides income diversification, more biodiverse systems can stabilize agroecosystem productivity through cross-scale mechanisms ranging from redundancy and facilitation in plant communities^41,53^, to creating habitat for natural enemies to promote pest suppression^12^. Conservation practices, in turn, can improve properties related to soil health and crop yield stability, such as soil organic matter and water retention^49,50^. However, the effects of increasing system diversity and ecological complexity by adding a trophic level (i.e., grazing animals) on the long-term stability of no-till cropping systems have not yet been studied with the same level of detail.

The primary goal of this study was to evaluate long-term yields and stability of ICLS yields and profitability compared to non-integrated systems under a range of environmental conditions and test their potential as a strategy for sustainable intensification. We hypothesized that increased biodiversity and ecological complexity created by crop-livestock integration in no-till systems improve yields while decreasing vulnerability of system yields and profitability to weather variation. We tested this hypothesis using a 16-year dataset from a long-term, no-till integrated soybean-beef cattle system in southern Brazil and measured the impacts of cover crop grazing at different intensities during the winter period on probability of high and low performance^13,15,44^, minimum and maximum yield potentials and stability of key crop, pasture, animal and whole-system outcomes using established metrics of stability^22,44–50^. Our results provide insight into the long-term stability of subtropical soybean systems performance and the potential of livestock integration to build up sustainability and resilience in agriculture.

## Methods

### Site description and experimental design

The experiment was established at Espinilho Farm, in the municipality of São Miguel das Missões, Rio Grande do Sul State, southern Brazil (28° 56′ 14″ S, 54° 20′ 52″ W, 465 m above sea level) in 2001. The region has a warm, humid subtropical climate (Cfa, Köppen classification system) with an average annual temperature of 18.6 °C and average annual precipitation of 1898 mm^54^. Temperature and precipitation during the experimental period analyzed here (2001–2016) were collected by a weather station located at the experimental site (Supplementary Fig. S1). Missing weather data points were estimated using linear regression with values from the nearest meteorological station as predictor (National Institute of Meteorology, Cruz Alta, 78 km from the study site 28° 36′ 12′′ S, 53° 40′ 25′′ W, 427 m a.s.l.). The soil in the experimental site is an Oxisol (Rhodic Hapludox)^55^, with clayey texture (540, 270 and 190 g kg^−1^ of clay, silt and sand, respectively) and a deep, well drained profile.

The area has been managed as no-till soybean [*Glycine max* (L.) Merr.] cropland since 1993. In 2001, 22 hectares of land began to be managed as an integrated soybean-beef cattle annual rotation with a mixture of black oat (*Avena strigosa* Schreb.) and Italian ryegrass (*Lolium multiflorum* Lam.) pastures grazed during the winter between soybean crops. Soybean was direct-seeded after the animals were removed from the experimental area, typically in November, and harvested after 142 ± 11 days. After soybean harvest (April–May), experimental plots were drill-seeded with black oats into the volunteer ryegrass sward from the previous winter, immediately followed by broadcast seeding of ryegrass to ensure successful establishment for both species in all treatments.

The experiment was established as a randomized complete block design with three replicates. Treatments consisted of four grazing intensities (intense, moderate, moderate-light and light) defined by contrasting sward heights under continuous stocking (10, 20, 30 and 40 cm, respectively) and an ungrazed control with the same pasture species used as winter cover crops. Plot areas were 0.1 ha for the ungrazed treatment and ranged from 0.8 to 3.6 ha for grazed treatments. Plots differed in area to reduce the number of animals required to maintain the target treatment heights, especially for shorter swards.

Fertilization rates and soybean cultivars changed according to recommendations over the years but were the same for all plots, including the ungrazed treatment. An average of 160 kg ha^−1^ urea (46% N) was applied yearly, split into two equal winter applications during the stocking period: (1) when pasture reached V3–V4 growth stage (i.e., plants with 3–4 fully expanded leaves on the main stem) and (2) just before animals entered the experimental plots, approximately 1 month after the first application. From 2001 to 2011, P and K (on average, 60 kg ha^−1^ P_2_O_5_ and 70 kg ha^−1^ K_2_O) were applied at soybean sowing. From 2012 to 2016, P and K (on average, 45 kg ha^−1^ P_2_O_5_ and 60 kg ha^−1^ K_2_O) were applied at pasture sowing to take advantage of the improved nutrient recycling provided by the grazing animals for primary production. The exact amount of fertilized applied each year was based on standard recommendations^56^ and soil analysis.

Grazing usually started in June–July, when average sward height reached 24 ± 4 cm (or 1485 ± 379 kg ha^−1^ of dry matter) and lasted 124 ± 16 days. To ensure that treatments remained close to their nominal targets (Supplementary Fig. S2), sward height was measured at 100 random points per plot every 15 days with a sward stick^57^. Three tester animals remained permanently in the plots over the stocking period and put-and-take animals were added or removed to adjust sward heights^58^. Average stocking rates used to maintain target sward heights throughout the stocking period were 376, 651, 948 and 1331 kg of live weight ha^−1^ for light to intense grazing. Experimental animals were crossbred Angus × Hereford × Nelore steers with initial body weight of 210 ± 23 kg and 12 months of age on average. Steers were weighed at the beginning and at the end of the stocking period after 12 h of fasting.

### Long-term data collection and variables studied

We assessed five key indicators of crop, pasture, animal and whole-system performance: (1) soybean grain yield; (2) total herbage production; (3) animal live weight gain; (4) human-digestible protein (HDP) production; and (5) profitability. “Year” in all analyses refers to the year when soybeans were sown. To avoid bias, specific years were removed from the analysis when data for one or more treatments were missing for a variable. Years 2001, 2003 and 2008 were excluded from the analyses of soybean yield, protein production and income. Years 2001, 2004, 2005, 2006, 2007 and 2012 were excluded from the analyses of herbage yield. Year 2012 was excluded from the analysis of animal production, protein production and income.

Soybean yield (kg of grains ha^−1^) was determined at full grain maturity at 13% moisture content. Total herbage production (kg of dry matter ha^−1^) was calculated as the sum of pasture herbage mass on the first grazing day and the daily herbage accumulation rates over the whole stocking period. Daily herbage accumulation rates were estimated every 28 days using grazing exclusion cages^59^, following a standard protocol described by Nunes et al.^60^. Steer live weight gain (kg of live weight ha^−1^) was calculated as the product of number of animals per hectare, average daily gain (kg of live weight steer^−1^ day^−1^) of the tester animals and number of grazing days of the stocking period.

We adopted human-digestible protein (HDP) as a metric to account for added production from the livestock component when comparing integrated to non-integrated systems. Livestock contributes to supplying human protein demand as much or more than crop production^39,61^ and do so by converting proteins from non-edible (grass) into edible forms. HDP is not intended as a comprehensive nutritional analysis; rather, it is an unbiased indicator of whole-system food production^62^. Total HDP production (kg ha^−1^) was calculated as the sum of protein from human-edible sources (i.e., animal and crop components of the system) multiplied by protein digestibility of the products (beef and soybeans)^61^. We estimated the protein content of a 350 kg live weight steer at the end of the stocking period as 19% of its body weight, based on National Research Council’s equations^63^. Soybean protein content was assumed to be 35% for a grain moisture content of 13%^64^.

We used gross profit (USD ha^−1^) as a metric of profitability, calculated as: (1) the difference between operational costs of soybean and cover crops and revenues from grain sales in the specialized system (Supplementary Table S1); and (2) the difference between costs of soybean, cover crops and livestock operations (Supplementary Table S2), opportunity cost of capital invested in beef cattle [calculated as the product of average stocking rate, cattle price and saving account interest rate equivalent to the average number of grazing days (~ 2% interest rate) according to the Central Bank of Brazil, Supplementary Table S2]^65^ and revenues from animal and grain sales in the integrated crop-livestock systems. Revenues were calculated using yearly market sale prices of beef cattle and soybean grains on November and April, respectively (Supplementary Table S3)^66,67^. Historic nominal prices were transformed into real values using the General Market Price Index (IGP-M) from the Getúlio Vargas Foundation (FGV), Brazil, using 2016 as the base year for the analysis^68^ and converted from Brazilian Reals (BRL) to U.S. Dollars (USD) using the exchange rates of the respective months^69^. We used steer live weight gain to calculate income from livestock, assuming similar price per unit mass of beef at purchase and sale. Annual costs of soybean production were obtained from Brazil’s National Supply Company for the study region^70^. Soybean costs were considered the same for all treatments, given similar crop management across experimental units. Annual costs of cover crop establishment, animal medicines and mineral supplementation for the period of 2002–2011 were obtained from the economic analysis done by Oliveira et al.^33^ in the same experimental protocol. For the period of 2012–2016 costs were estimated using linear regression. Both costs and revenues were detrended prior to the calculation of annual profits.

### Statistical analysis

All statistical analyses were performed in R (version 3.6.1)^71^. Long-term crop, pasture, animal and whole-system mean yields were analyzed using the *lme4* package for mixed linear models^72^ with treatments as fixed effects and years, blocks and plots within blocks as random effects (y ~ factor(year) * treatment + (1|block/plot)). Yield trends over the 16 years were analyzed using linear mixed-effects models with treatments and years as fixed effects and blocks and plots within blocks as random effects (y ~ year * treatment + (1|block/plot)). Analysis of variance (ANOVA, Supplementary Tables S4 and S5) was performed and when significant effects were detected, treatment means were compared with Tukey test at 95% confidence level using the *emmeans*^73^ and *lmerTest*^74^ packages. Residuals of all analyses were visually checked for homogeneity of variance and normality was tested with quantile–quantile plots using the R *car* package^75^. When the residuals were not homogeneous or the distribution was not normal, data were log or square root transformed as appropriate.

### Yield stability analysis

We assessed stability of production (soybean yield, total herbage production, animal live weight gain, human-digestible protein) and profitability using four different metrics of stability: (1) yield range, which is the maximum amplitude between minimum and maximum yield values in a time series^44,45^; (2) coefficient of variation and (3) standard deviation^21,44,45^; and (4) Finlay and Wilkinson’s stability metric (FW) derived from the linear regression of treatment yield on the mean yield of the location/year, or Environmental Index (EI)^44–49^. Regression of detrended yield on EI, also called adaptability analysis^47^, can assess stability or treatment-specific effect across a range of environments^49^. Based on regression of detrended yield on EI, stable systems are those with smaller slope (less sensitive to changes in environment).

Yield range was calculated as the difference between the highest and the lowest yields for each variable over the experimental period. Coefficient of variation, standard deviation and FW regressions were calculated using detrended data. Detrending removed long-term linear trends potentially generated by treatments in order to only consider variability of the residuals around the mean of each treatment due to transient environmental conditions. Data were detrended by removing treatment effects and treatment-specific linear temporal trends using the residuals of the linear model y ~ year * treatment. The overall average of the response variable was added to the residuals to get intuitively more understandable values (addition of the same constant to all values does not affect relevant statistical results).

Detrended data were analyzed as a function of the Environmental Index (EI) for each year and treatment with the following model: detrended y ~ EI * treatment. EI was calculated as the average yield of all treatments for each year, so that the highest and lowest EI indicated the year of highest and lowest system performance respectively. FW regression slopes were calculated and compared using simultaneous general linear tests with the R *multcomp* package^76^.

Yield range, coefficient of variation and standard deviation were analyzed as a function of treatment and block (y ~ treatment + block) and when significant differences were detected, treatment means were compared with Tukey test at 95% confidence level (⍺ = 0.05) using the R *agricolae* package^77^.

Treatments were ranked from the lowest (i.e., greatest stability, rank #1) to the highest value (i.e., lowest stability, rank #5) for each stability metric regardless of the statistical significance. The overall stability of each system output was ranked based on mean stability rank for the four stability metrics, such that treatments with higher overall ranks indicated higher stability of yield or profitability.

### Minimum and maximum yield potentials

Minimum and maximum yield potentials were calculated based on predicted responses for the smallest and largest observed EI values for each studied indicator^44,49,50^. Treatment effects on minimum and maximum yield potentials were tested with Tukey test at 95% confidence level through the equation $HSD=q{\sqrt{2 SE} }$, where *HSD* is Tukey’s honest significant difference,* q* is the studentized range statistic obtained using the ‘qtukey’ function from R *stats* package^71^, and *SE* is the standard error of the mean for the studied variable.

### Downside risk and probability of high performance

To determine the probability of extreme yield events over the given range of environmental conditions (EI), we modelled probability distributions of each treatment’s detrended data using the ‘density’ function in R (Supplementary Code S1, adapted from Gaudin et al.^13^). Treatment distributions were compared to a randomized distribution created by bootstrapping data and ignoring treatment effects. Downside risk and probabilities of high performances were defined as estimated probabilities of achieving results below the 10th percentile and above the 90th percentile, respectively, for each of the studied indicators. 5000 randomizations were sufficient to stabilize the *p* values for every system output. Treatment effects on the downside risk or probability of high performance were identified when observed results were significantly different from the randomized distribution at the 95% confidence level beyond the determined percentiles.

## Results

### Mean yields and trends

Soybean yields were not affected by winter grazing of cover crops, regardless of the grazing intensity (*p* = 0.375, Table 1). Total herbage production increased with increasing sward height (*p* < 0.001, Table 1) but remained low in the ungrazed treatment. Steers’ live weight gain per unit area increased with grazing intensity (*p* < 0.001, Table 1). Addition of cattle to the system increased total human-digestible protein production by up to 13% (*p* = 0.065, Table 1). Profitability in the two highest grazing intensities was 38% greater than in the two lowest ones, and 112% greater than in the ungrazed treatment (*p* < 0.001, Table 1).

**Table 1:** Mean yields and stability parameters of a long-term (2001–2016) soybean system integrated with livestock at different grazing intensities or left ungrazed in the winter period.

Indicator	Treatment	Mean yield	Stability parameters				Overall rank
			Yield range	Coefficient of variation (%)	Standard deviation	FW slope	
Soybean yield (kg grain ha^−1^)	G10	2882.58	4020.60 (3)	40 (3)	1157.72 (4)	0.99 (2)	3
	G20	2857.27	3993.37 (2)	39 (2)	1127.47 (2)	0.98 (1)	1.8
	G30	2835.15	4026.83 (4)	40 (3)	1115.64 (1)	0.98 (1)	2.3
	G40	3086.35	4030.53 (5)	38 (1)	1157.51 (3)	1.02 (3)	3
	UG	2974.50	3797.03 (1)	46 (4)	1279.53 (5)	1.04 (4)	3.5
Total herbage production (kg DM ha^−1^)	G10	6493.02 b	5382.73 (2)	26 (3)	1678.63 (1)	0.87 (2)	2
	G20	7447.46 ab	6445.77 (4)	28 (4)	2119.21 (4)	1.08 (4)	4
	G30	7735.80 a	7023.67 (5)	29 (5)	2202.73 (5)	1.21 (5)	5
	G40	8118.69 a	6074.67 (3)	24 (1)	1949.07 (3)	0.96 (3)	2.5
	UG	6859.84 ab	5079.05 (1)	25 (2)	1733.51 (2)	0.81 (1)	1.5
Live weight gain (kg LW ha^−1^)	G10	509.92 a	348.23 a (4)	18 (2)	93.75 a (4)	1.66 a (4)	3.5
	G20	428.41 b	270.57 ab (3)	19 (3)	80.35 ab (3)	1.57 a (3)	3
	G30	310.83 c	212.27 ab (2)	18 (2)	56.23 bc (2)	0.59 b (2)	2
	G40	183.16 d	135.67 b (1)	16 (1)	28.74 c (1)	0.18 b (1)	1
Human-digestible protein production (kg HDP ha^−1^)	G10	780.55	1121.66 (5)	36 (1)	320.14 (4)	0.99 (2)	3
	G20	765.09	1111.16 (4)	36 (1)	314.16 (3)	0.99 (2)	2.5
	G30	720.54	1095.90 (3)	37 (2)	304.94 (1)	0.98 (1)	1.8
	G40	749.94	1094.92 (2)	37 (2)	313.69 (2)	1.00 (3)	2.3
	UG	692.29	1036.59 (1)	46 (3)	349.31 (5)	1.03 (4)	3.3
Profitability (USD ha^−1^)	G10	940.25 a	1679.39 (4)	51 b (1)	509.27 (4)	1.06 (4)	3.3
	G20	842.81 a	1849.11 (5)	57 b (2)	513.92 (5)	1.10 (5)	4.3
	G30	691.24 b	1467.58 (1)	58 b (3)	435.60 (2)	0.94 (2)	2
	G40	598.09 b	1475.99 (2)	66 b (4)	430.82 (1)	0.92 (1)	2
	UG	419.84 c	1621.14 (3)	106 a (5)	490.09 (3)	0.98 (3)	3.5

G10: intense grazing (10 cm sward height); G20: moderate grazing (20 cm sward height); G30: moderate-light grazing (30 cm sward height); G40: light grazing (40 cm sward height); UG: ungrazed cover crop. FW slope represents the Finlay and Wilkinson regression slope. Numbers in parentheses rank the treatments for each variable within each column. Different letters in the column represent significant differences among treatments according to the Tukey test (⍺ = 0.05).

All variables, except for total herbage production, presented an increasing linear trend over time (Supplementary Fig. S3, Supplementary Table S5). None of the linear trends were significantly affected by treatments, as indicated by the absence of treatment by year interactions (Supplementary Table S5). When year was included as a factor (categorical variable) in the model, there was a significant treatment by year interaction for total herbage production and live weight gain (Supplementary Table S4). However, we were unable to detect a clear pattern in the interactions.

### Yield stability

**Figure 1:**
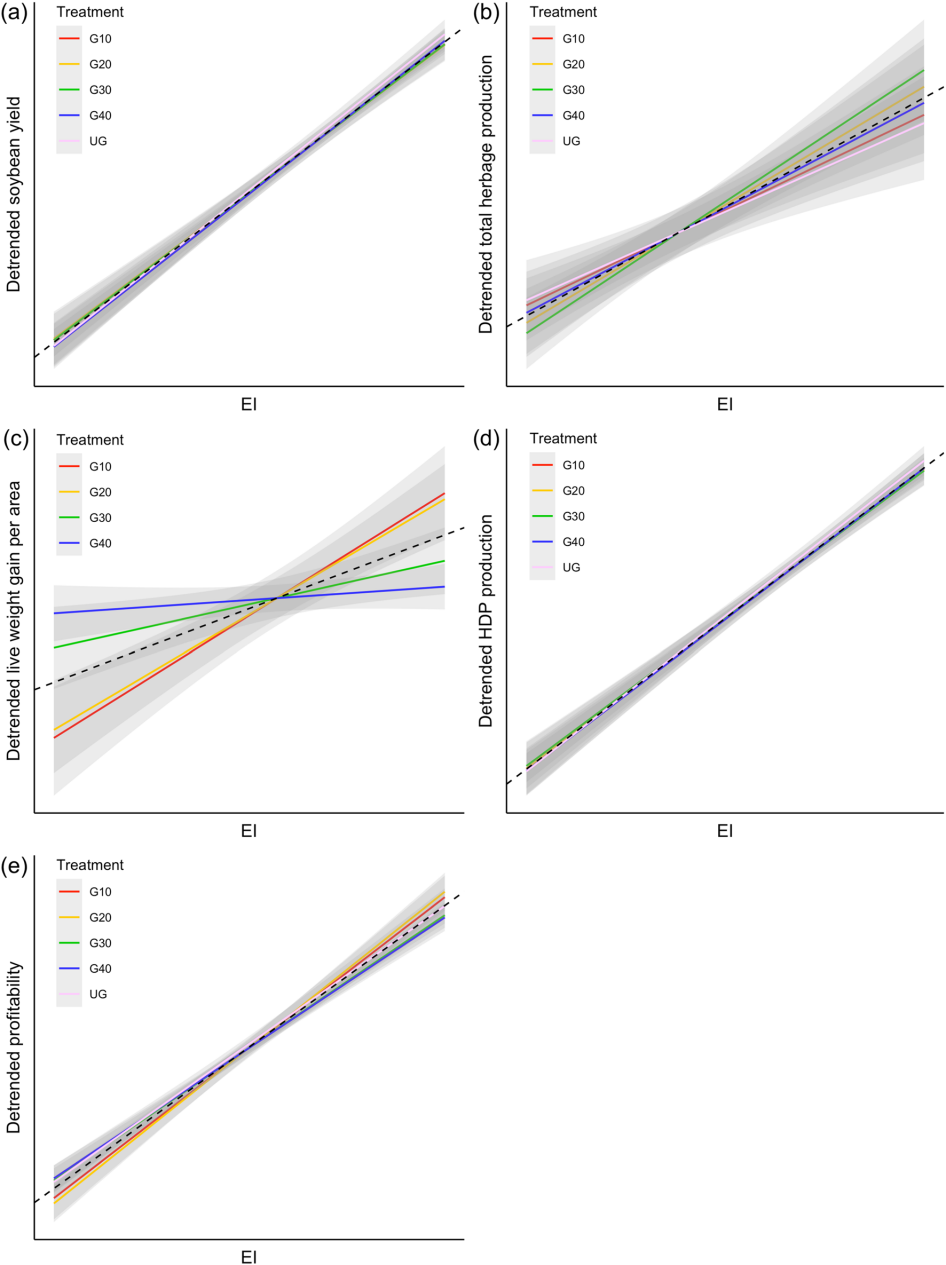
Yield stability of (**a**) soybean yield (kg grain ha^−1^), (**b**) total herbage production (kg dry matter ha^−1^), (**c**) animal live weight (LW) gain (kg LW ha^−1^), (**d**) human-digestible protein (HDP) production (kg HDP ha^−1^) and (**e**) profitability (USD ha^−1^) of soybean systems integrated with different levels of cattle grazing during the winter period. Environmental index (EI) was calculated as the yearly mean detrended yield. Dashed lines are the regression of detrended yields against the EI without treatment effects. G10: intense grazing (10 cm sward height); G20: moderate grazing (20 cm sward height); G30: moderate-light grazing (30 cm sward height); G40: light grazing (40 cm sward height); UG: ungrazed cover crop. Smaller slopes indicate greater yield stability.

Soybean yield was the most stable when the pasture phase was managed at moderate grazing intensities (G20 and G30) according to the overall stability rank (Table 1). Ungrazed (UG) and lightly grazed (G40) treatments were more sensitive to the environmental gradient than more intensively grazed treatments (FW slopes > 1, Fig. 1a, Table 1), indicating lower stability. The ungrazed treatment presented the narrowest yield range but was ranked worst in all the other stability metrics, making it the least favorable to soybean yield stability (Table 1). Intense (G10) and light grazing (G40) were similar and intermediate in overall stability.

Conversely, total herbage production was the least stable under moderate grazing intensities, with G30 and G20 ranking fifth and fourth, respectively, in all stability metrics (Table 1). Both treatments were more responsive to changes in Environmental Index (Fig. 1b). The UG control presented the most stable herbage production over the years, ranking first in FW slopes and yield range, and second in CV and standard deviation, followed by G10 and G40 (Table 1).

Increasing grazing intensity reduced the overall stability of live weight gain (Table 1). Light grazing (G40) ranked first for all stability metrics for live weight gain and, along with G30, was significantly more stable than G10 and G20 to the environmental gradient (*p* < 0.05, Fig. 1c, Table 1).

Both human-digestible protein (HDP) production and profitability showed greater stability when pastures were grazed at moderate to light intensities (G30 and G40), while either over-intensification or the absence of grazing decreased system stability (Table 1). The UG control had a 26% and 83% higher CV for HDP production and profitability, respectively, than the grazed treatments and was the only stability metric showing statistical significance for profitability (*p* < 0.05, Table 1). FW slopes for HDP production followed the same trends as soybean yields, with greater slopes (> 1) for the G40 and UG treatments (Fig. 1d, Table 1). Profitability, in turn, trended together with live weight gains, with lower FW slopes for G30 and G40 and greater slopes for G10 and G20 (Fig. 1e, Table 1). Differently from live weight gains, however, G20 was less stable than G10 in all stability metrics except for CV (Table 1). The combination of prices and live weight gains might have been the reason why G10 and G20 switched positions in the profitability rank.

### Downside risk and minimum yield potentials

**Figure 2:**
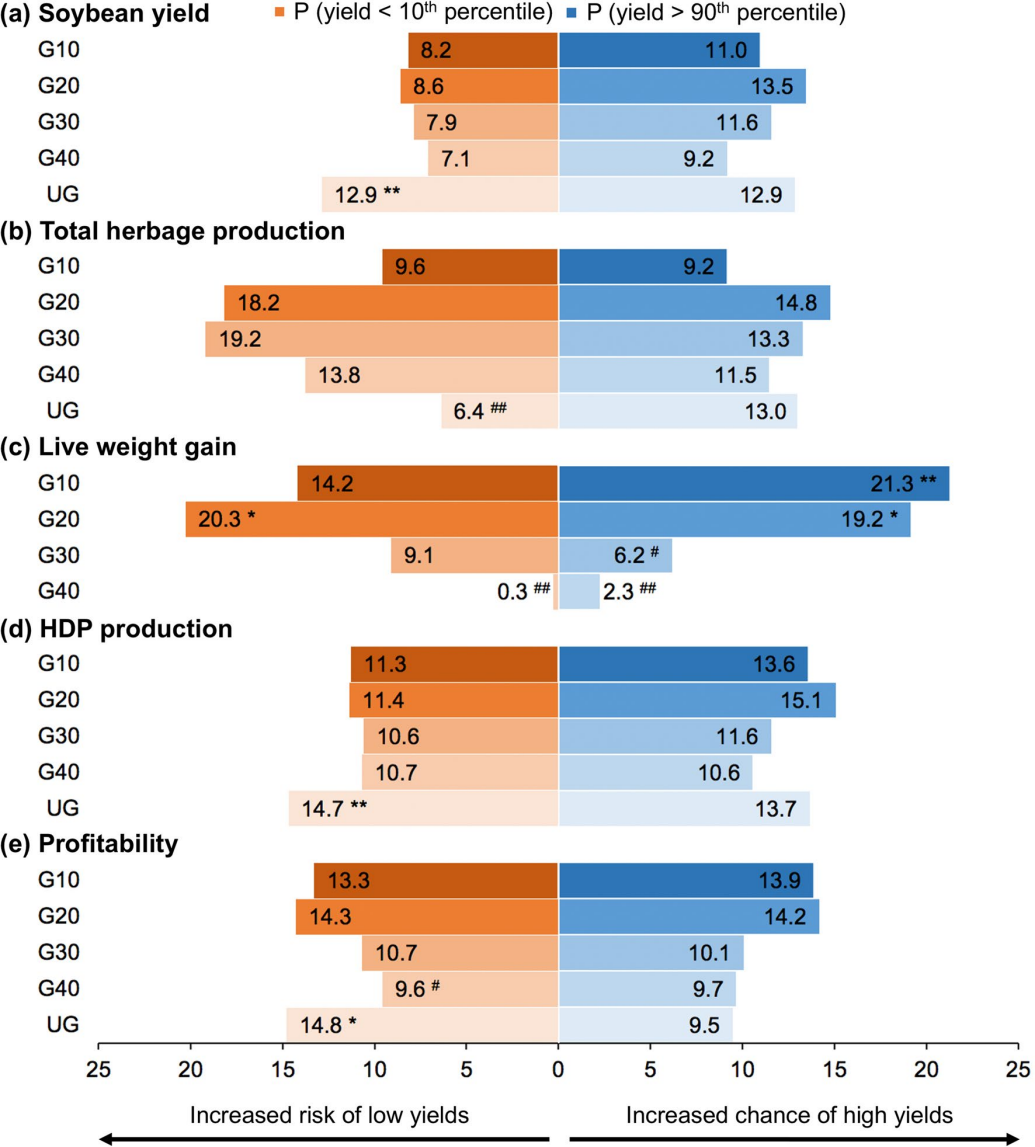
Effect of grazing intensity on the probability of obtaining high and low (**a**) soybean yield (kg grain ha^−1^), (**b**) total herbage production (kg dry matter ha^−1^), (c) animal live weight (LW) gain (kg LW ha^−1^), (**d**) human-digestible protein (HDP) production (kg HDP ha^−1^) and (**e**) profitability (USD ha^−1^) of soybean systems integrated with different levels of cattle grazing during the winter period in southern Brazil. Shown are the probabilities of yielding below the 10th percentile (orange bars) or above the 90th percentile (blue bars). Statistically significant treatment effect was identified for higher probability of high/low yields at the 95% (*) or 99% (**) confidence level and for lower probability of high/low yields at the 95% (^#^) or 99% (^##^) confidence level. G10: intense grazing (10 cm sward height); G20: moderate grazing (20 cm sward height); G30: moderate-light grazing (30 cm sward height); G40: light grazing (40 cm sward height); UG: ungrazed cover crop.

**Figure 3:**
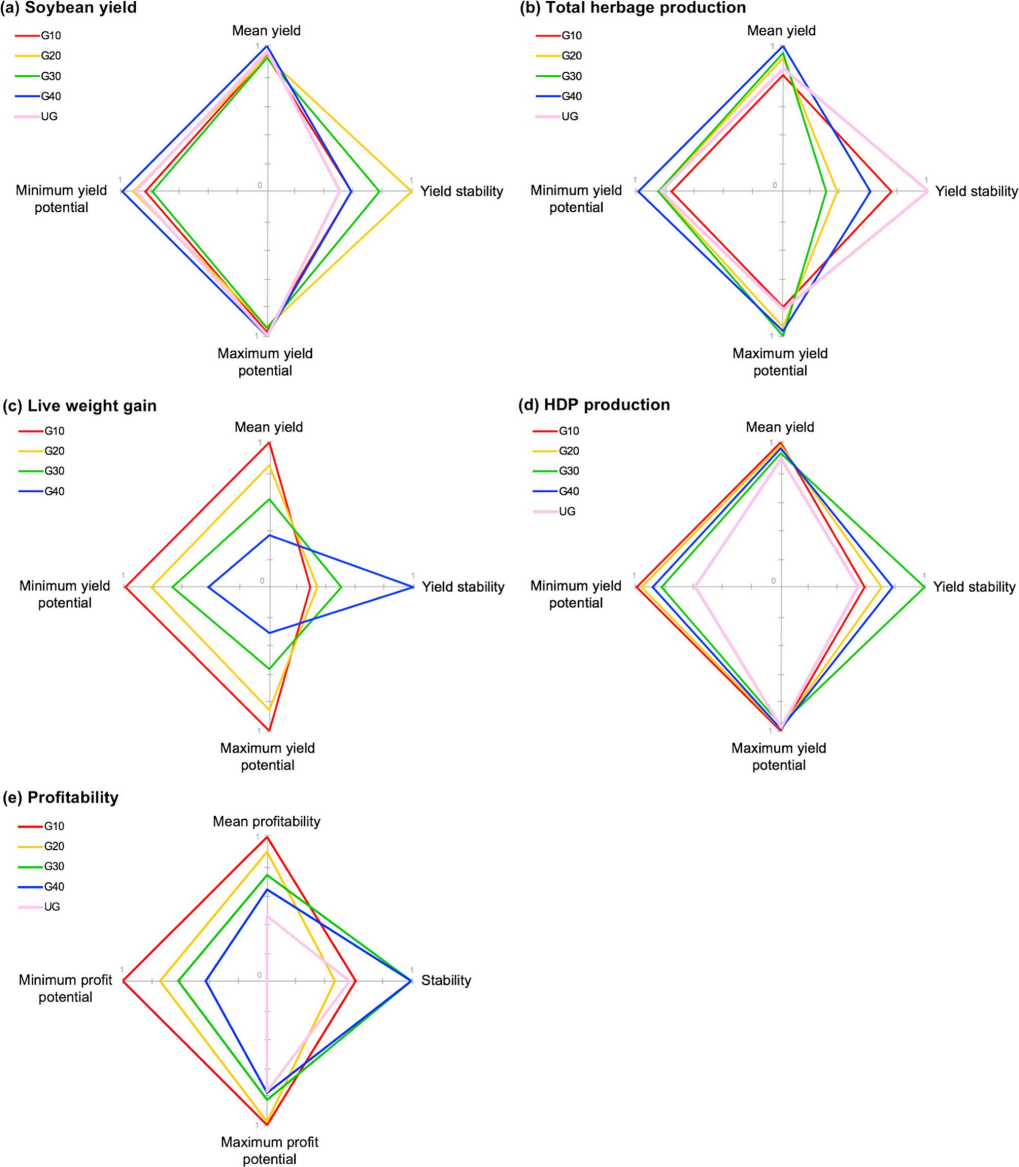
Tradeoffs between performance and stability of (**a**) soybean yield (kg grain ha^−1^), (**b**) total herbage production (kg dry matter ha^−1^), (**c**) animal live weight (LW) gain (kg LW ha^−1^), (**d**) human-digestible protein (HDP) production (kg HDP ha^−1^) and (**e**) profitability (USD ha^−1^) of soybean systems integrated with different levels of cattle grazing during the winter period in southern Brazil. Values represent standardized ratio to the maximum value for each metric. Yield stability is the average rank of four stability metrics (Table 1).

The absence of grazing (UG) significantly (1) increased the downside risk for soybean yield (⍺ = 0.01, Fig. 2a) without significantly impacting the minimum yield potential (Table 2); (2) increased risks of obtaining low HDP production (⍺ = 0.01, Fig. 2d); (3) increased risks of financial loss (⍺ = 0.05, Fig. 2e); and (4) had the lowest minimum profitability (− 215.42 USD ha^−1^, *p* < 0.05, Table 2). Both downside risk and minimum yield potential can be used as proxies of system resistance, since they represent the ability of a system to avoid crop failure or financial loss under stressful environmental conditions^44^. That said, livestock integration increased system resistance to financial loss by 81% in the lightest grazing intensity (G40) and up to 188% in the highest grazing intensity (G10) compared to the ungrazed control in the harshest environmental conditions (*p* < 0.05, Table 2). Despite minimum HDP production not being significantly different among treatments, it was 55% greater in grazed treatments compared to UG and up to 69% greater than UG in the highest grazing intensity.

**Table 2:** Minimum and maximum yield potentials of a long-term (2001–2016) soybean system integrated with livestock at different grazing intensities or left ungrazed in the winter period.

Indicator	Treatment	Minimum yield potential	Maximum yield potential
Soybean yield (kg grain ha^−1^)	G10	509.43	4925.05
	G20	558.09	4746.89
	G30	479.18	4784.52
	G40	605.47	5049.36
	UG	548.67	5026.09
Total herbage production (kg DM ha^−1^)	G10	4798.73	9271.93
	G20	5347.92	10,891.05
	G30	5396.34	11,572.91
	G40	6252.24	11,179.98
	UG	5287.69	9445.18
Live weight gain per area (kg LW ha^−1^)	G10	399.79 a	593.43 a
	G20	327.30 ab	507.20 a
	G30	270.06 b	338.66 b
	G40	170.32 c	190.26 c
Human-digestible protein production (kg HDP ha^−1^)	G10	234.54	1267.77
	G20	225.82	1250.97
	G30	193.21	1211.53
	G40	207.71	1254.28
	UG	139.02	1241.88
Profitability (USD ha^-1^)	G10	189.04 a	1888.02 a
	G20	84.62 ab	1845.45 ab
	G30	35.57 ab	1559.39 bc
	G40	− 40.40 b	1467.44 c
	UG	− 215.42 c	1447.02 c

G10: intense grazing (10 cm sward height); G20: moderate grazing (20 cm sward height); G30: moderate-light grazing (30 cm sward height); G40: light grazing (40 cm sward height); UG: ungrazed cover crop. Different letters in the column represent significant differences among treatments according to the Tukey test (⍺ = 0.05).

Conversely, UG presented a lower risk of low herbage production (⍺ = 0.01, Fig. 2b) despite no statistical differences in minimum yield potential (Table 2). G20 and G30 had higher probability of low herbage production (18 and 19%, respectively), but were not different from the random distribution (⍺ = 0.05, Fig. 2b). G20 probability of low live weight gain was significantly higher (⍺ = 0.05). G40 presented significantly lower downside risk for live weight gain (⍺ = 0.01, Fig. 2c) but also a significantly lower minimum live weight gain potential (⍺ = 0.05, Table 2).

### Probability of high performance and maximum yield potentials

Treatment effect on the probability of high performance was larger for live weight gains than for the other variables. High (G10) and moderate (G20) grazing intensities significantly increased the chance of obtaining live weight gains above the 90th percentile (⍺ = 0.01 and ⍺ = 0.05, respectively, Fig. 2c). Conversely, moderate-light (G30) and light (G40) grazing intensities reduced the chance of high live weight gains (⍺ = 0.05 and ⍺ = 0.01, respectively, Fig. 2c) and maximum yield potentials relative to G10 and G20 (Table 2).

We observed greater maximum profitability potential in G10 and G20 than in the UG control (1866.73 average vs. 1447.02, a 29% increase, *p* < 0.05, Table 2), but probability of high performance was not affected (Fig. 2e). No changes in probability of high performance were detected for soybean yield, total herbage production and HDP production. Likewise, maximum yield potentials were not statistically different between treatments (Table 2), despite the important difference in pasture dry matter production from G10 and UG to moderate to light grazing intensities (G20, G30 and G40) that ranged from 1445.87 kg DM ha^−1^ (G20 vs. UG) to 2300.98 kg DM ha^−1^ (G30 vs. G10).

The most important findings of our study were: (1) grazing did not impair subsequent soybean yields regardless of grazing intensity, but moderate grazing intensities favored long-term yield stability (Fig. 3a); (2) herbage production was more stable over the years but significantly lower under heavy grazing and in the absence of grazing (Fig. 3b); (3) live weight gains were generally greater but less stable at higher grazing intensities (Fig. 3c); (4) grazing at moderate to light intensities increased the stability of HDP production, while over-intensification and absence of grazing increased system vulnerability to environmental oscillations (Fig. 3d); and (5) livestock integration under lighter grazing intensities provided more stable profits over time, but risk of financial loss reduced and overall system profitability increased with grazing intensity (Fig. 3e).

## Discussion

Integrated crop-livestock systems (ICLS) are proposed as one possible strategy towards the sustainable intensification of food systems^2,23,29,30^. In a context of climate change and increased environmental pressure, stability of agricultural systems performance—not just performance per se—needs to be evaluated to prioritize management strategies with the greatest adaptive gains. Mining data from long-term trials provides opportunities to comprehensively assess the performance and stability of key crop, pasture, animal and whole-system indicators when livestock is integrated into specialized cropping systems.

Grazing did not impair soybean grain yields regardless of grazing intensity, but moderate grazing intensities favored soybean long-term yield stability (Fig. 3a). Our analysis of soybean yields supports previous studies showing that grazing is not detrimental to crop productivity^37,78,79^. The impact of livestock on subsequent crop yields has long been a concern, mainly due to potential soil compaction caused by animal trampling, consumption of cover crop biomass and nutrient export when animals are removed from the system^23,78^. Results from literature on ICLS have shown everything from decreases in subsequent crop yield^80^, to no effect^37,78,79^ and even increases^23,30,81^. In our systems, grazing is combined with low disturbance (i.e., no-till) which may help mitigate potential negative impacts such as soil compaction. When conservation agricultural practices are used and grazing is well-managed (i.e., assuming no overgrazing or abnormally wet years), effects on soil physical attributes such as increased soil density have been shown to be transient, restricted to soil surface, and of limited impacts on yields^78^.

Our study provides the first evidence of grazing-induced long-term yield stability in no-till soybean systems where crops and livestock were integrated under moderate grazing intensity (Fig. 3a). Furthermore, our risk analysis has shown that the absence of grazing increases the risk of yielding below the 10^th^ percentile in unfavorable years (Fig. 2a), despite greater litter amounts covering soil in ungrazed plots^60^. The underlying processes of increased yield stability with moderate grazing may be associated with increased biological diversity and ecological interactions created by livestock integration. Properties associated with the maintenance of soil functions and crop stability such as soil aggregation^32^, microbial diversity^36,82^ and ratios of beneficial over detrimental soil nematodes^82^ were shown to be improved by moderate grazing in previous studies at this experimental site and may have provided better growing conditions for the soybean crop in stressful years. Moderate grazing intensities enhance root growth, exudation and turnover which, combined with manure deposition, can directly benefit soil aggregation and microbial activity and diversity^32^. This in turn can lead to greater soil physical stabilization, organic matter accumulation^83,84^ and nutrient cycling^84^. These soil health benefits, including more biodiverse soil communities, may be particularly relevant to maintain soil functioning under stress as shown in other systems^85,86^ and potential core mechanisms underlying crop yield stability.

Total herbage production increased with increasing sward height but remained low in the ungrazed treatment, and was the least stable under moderate grazing intensities, demonstrating a possible trade-off between yield and stability in forage crops (Fig. 3b). No grazing (UG) and heavy grazing (G10) treatments were more stable but produced significantly less forage over the years (Table 1). Moderate grazing intensities created more responsive forage growth to better environmental conditions and along with light grazing (G40) were able to produce ~ 11 tons DM ha^−1^, while G10 and UG reached less than 10 tons DM ha^−1^ even in the best environment (Table 2). Lower stocking rates in G40 and UG favored the maintenance of target sward heights (and consequently leaf area index) in dry years, so that daily herbage accumulation rates in these treatments were less affected by poor environmental conditions. These results support long established plant–herbivore models^87^ and the existence of two stable steady-states between vegetation growth and animal consumption in grazing lands: a low-productivity stable equilibrium at low plant biomass (G10), and a high-productivity stable equilibrium at high plant biomass (somewhere between G40 and UG). Moderate grazing (G20 and G30) provided a mid-range unstable state at which pasture growth is high, but herbage mass and accumulation rates are more easily affected by disturbances (e.g., weather fluctuations, fertilization or grazing itself), thus requiring more frequent adjustments of stocking rate to keep sward heights close to the nominal targets^87^.

In the absence of grazing, forage yields presented a lower risk of low production in unfavorable years (Fig. 2). Keeping a dense layer of residual biomass on the soil surface in no-till systems (during winter as cover crop/pasture and after winter, as straw) improves soil water retention^37^ and protects soil from erosion^88^ and weed outbreak^89^ with potential benefits to crops in rotation. For this reason, crop-livestock integration is seen by many farmers as detrimental to no-till systems. However, prior research at this site showed no direct impacts of greater litter mass on crop yields in the ungrazed system^37^. On the other hand, the greater herbage production under moderate to light grazing intensities and the reduced probability of low forage yields in the ungrazed system found in our study may help explain the increased soil carbon stocks found by previous authors in areas managed under these approaches compared to intensely grazed areas after a decade of crop-livestock integration at this site^38^. Previous studies in humid continental climate of the US have shown that agronomic practices able to increase soil water holding capacity and organic matter can buffer yield volatility of rainfed maize^49^, suggesting that our results may apply to different agroecosystems around the world.

The linear increase of live weight gains per unit area (Table 1) with grazing intensity is consistent with previous studies and can be attributed to increased stocking rate required to keep pasture at target sward heights^90^. Constraints in animal dry matter intake when forage allowances are limiting could result in a quadratic response of live weight gain, with greater gains associated with moderate grazing intensities^91,92^. The shortest sward height used in our study in fact limits the intake^93^ and consequently the individual live weight gains^90,93^, but it was not restrictive enough to show the quadratic pattern when results were expressed on a per area basis because greater stocking rate compensated the decrease in individual performance.

Our analysis showed a clear trade-off between yields and stability of live weight gains (Fig. 3c). Live weight gains were generally greater, but less stable at higher grazing intensities. Although pasture growth is less stable and requires more frequent stocking rate adjustments under moderate grazing intensities, more intense stocking rate adjustments are required at the extremities of the grazing intensity gradient. In other words, the closer to a stable state, the stronger the push (i.e., addition or removal of animals) in the opposite direction required to shift states will be^87^. In our case, this was translated as a strong removal of animals from the plots when swards got too short to allow pasture regrowth in higher grazing intensities, which probably resulted in less stable live weight gains. Besides being less stable, literature also shows that higher grazing intensities lead to greater greenhouse gas emissions, especially methane^93^. Thus, to sustainably intensify ICLS, a ‘conciliatory stocking rate’^90,94^ able to achieve high animal yields and overall system stability while keeping low environmental footprint should be pursued.

Intensification of ruminant production in the last decades has increased protein production per area of land use, but primarily as a result of increased use of feed concentrates and human-edible nutrients in developed countries^10,39^. However, addressing the ability of a system to sustainably increase food production must consider the quality of food produced for human nutrition as well as the ability of this system to produce food from human inedible resources^39^. Grazing at moderate to light intensities increased HDP production and stability, while over-intensification and absence of grazing increased system vulnerability to environmental oscillations (Fig. 3d). Ungrazed cover crops represented a risk to food production in unfavorable years (Fig. 2d), since low soybean protein yields are not buffered by livestock protein yields as in integrated systems. By comprising protein from both crop and animal components of the system, our HDP analysis can be used as a measure of land-use efficiency^61^. Despite lacking statistical significance, grazing improved land-use efficiency by up to 13% due to the contribution of grass-based beef, an animal-derived protein of higher quality in human nutrition metrics than plant derived proteins^39^.

The greater profitability of integrated systems, particularly in heavier grazing intensities (G10 and G20, Fig. 3e, Table 1), was similar to results from a previous study at this site^33^ but differs in the magnitude of the results. While we observed profits 38% greater in the two highest grazing intensities (G10 and G20) compared to the two lowest ones (G30 and G40), and 112% greater than in UG treatment, Oliveira et al.^33^ found 27% and 100% increases (averages of 669, 526 and 334 USD ha^−1^, respectively). This difference might be explained by international meat prices, which raised steadily during the period comprised by their study (2001–2011) and remained relatively stable at a higher level after that^95^. Furthermore, two major droughts occurred during their study period and severely affected soybean yields and profitability of the systems. Moreover, soybean yields kept trending upwards from 2011 to 2016 (Supplementary Fig. S3). Another possible explanation is that those authors used the nominal purchase and sale prices practiced by the farmer at every beginning and end of stocking seasons, which might have led to lower profits because purchase prices per kg of yearling steers are usually higher than sale prices of steers at the end of the fattening period. Although one could argue that their method is more realistic than ours, we consider our method more reliable because it disregards potential benefits or disadvantages faced by the farmer when trading the animals over the years.

The decrease in stability of whole-system profits with the over-intensification or the absence of grazing was consistent with HDP production, with G30 and G40 being the most stable treatments (Fig. 3e, Table 1). However, while stability of HDP production was the lowest in the absence of grazing (UG), profits were less stable in G20 followed by UG and G10 according to the overall rank (Table 1). Considering our ranking criterion, this outcome was a result of G10 and G20 ranking 4th and 5th in every stability metric except for CV, in which they ranked 1st and 2nd (Table 1). The difference of CV to the other metrics is that it brings information of the variability relative to the mean. Therefore, according to the CV, livestock integration reduced the variability relative to the mean profit compared to UG regardless of grazing intensity, but when it comes to pure variability G10 and G20 were the least stable treatments.

These results countered our expectation that stability of profits would increase with stocking density, since literature suggests crops and livestock markets are uncorrelated, which would work as a buffer against climate and price fluctuations^28,34^. However, our results arise within the positively correlated beef cattle and soybean market prices in Rio Grande do Sul State during the experimental period (r = 0.44, 95% confidence interval = 0.30–0.56, *p* < 0.001, Supplementary Fig. S4a). Brazilian market prices of soybeans and cattle were also highly correlated in the last 2 decades (r = 0.88, 95% confidence interval = 0.87–0.88, p < 0.001, Supplementary Fig. S4b)^96,97^. Thus, while extra income from livestock might have buffered profit oscilations in G30 an G40 compared to UG (mainly through increasing system resistance in less optimal environments, Table 2), outstanding system performance in optimal environmental conditions (see maximum yield potential, Table 2) increased regression slopes and interannual variability in G10 and G20, resulting in the lower stability observed for these treatments (Table 1). Furthermore, the significantly higher risk of yielding below the 10th percentile (Fig. 2e) and the lower minimum profitability potential in UG (Table 2) represent a riskier farm portfolio, while animal production in grazed treatments provide a mean to smoothing farm incomes in poor crop production years (Table 2). These findings highlight the importance of using multiple metrics for studies associating system performance and long-term stability.

In conclusion, our data suggest that livestock integration into specialized soybean systems under moderate to light grazing intensities benefits whole system stability to environmental variability and confirm that grazing does not impair subsequent soybean yields in annual soybean-pasture rotations. Instead, it reduces the chance of crop failure in unfavorable years. Moreover, while livestock integration under lighter grazing intensities provides more stable profits over time, economic risk reduction and overall system profitability increase with grazing intensity, showing that probability and nature of win–win outcomes is a matter of management. Our results likely apply to other ICLS designs, but best pasture management remains paramount to achieve benefits and reduce potential tradeoffs. Our study also highlights the importance of long-term experimental protocols to understand complex temporal system responses such as yield stability and improve predictions and adaptation to climate change. Questions remain regarding what mechanisms are driving these results, especially for grazing-induced soybean yield stability, but intensification of ecological processes likely plays a pivotal role.

## Supplementary Information

Supplementary Information 1.

Supplementary Information 2.

## Data Availability

The datasets generated during and/or analyzed during the current study are available from the corresponding author on reasonable request.
