# Supplementary material for: Livestock integration into soybean systems improves long-term system stability and profits without compromising crop yields
Source: Sci Rep. 2021 Jan 18;11:1649. doi: 10.1038/s41598-021-81270-z (PMC7813827; doi:10.1038/s41598-021-81270-z)
Supplement: Supplementary file 1 — Supplementary Information 1. [file 41598_2021_81270_MOESM1_ESM.pdf]

Supplementary information for:

Livestock integration into soybean systems improves long-term system stability and profits without compromising crop yields

Pedro A. de A. Nunes[1,*], Emilio A. Laca[2], Paulo C. de F. Carvalho[1], Meng Li[2], William de Souza Filho[1], Taise R. Kunrath[1], Amanda P. Martins[3] and Amélie C. M. Gaudin[2]

[1]Department of Forage Plants and Agrometeorology, Federal University of Rio Grande do Sul, Porto Alegre, RS 91540-000, Brazil

[2]Department of Plant Sciences, University of California - Davis, Davis, CA 95616, USA

[3]Department of Soil Science, Federal University of Rio Grande do Sul, Porto Alegre, RS 91540-000, Brazil

[*]Corresponding author: pedro_nuness@hotmail.com

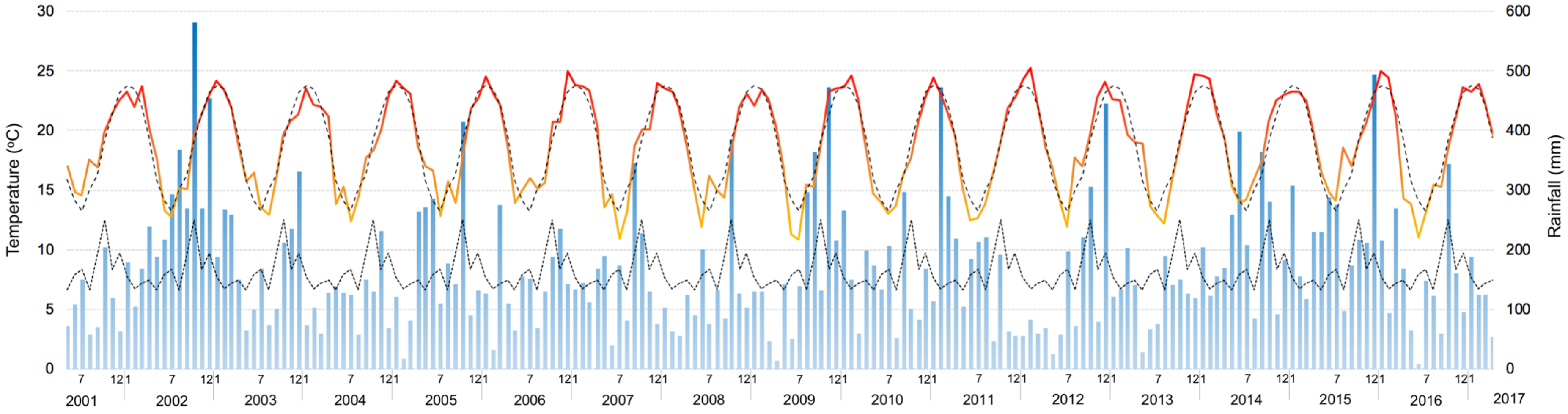

Supplementary Fig. S1. Monthly averaged environmental conditions at the experimental site (São Miguel das Missões, Rio Grande do Sul State, Brazil) for the period of May 2001 to April 2017. Orange continuous line shows air temperature (ºC) and blue bars show rainfall (mm). Dashed, black lines show the historical monthly averages for air temperature (top) and rainfall (bottom) for the experimental period.

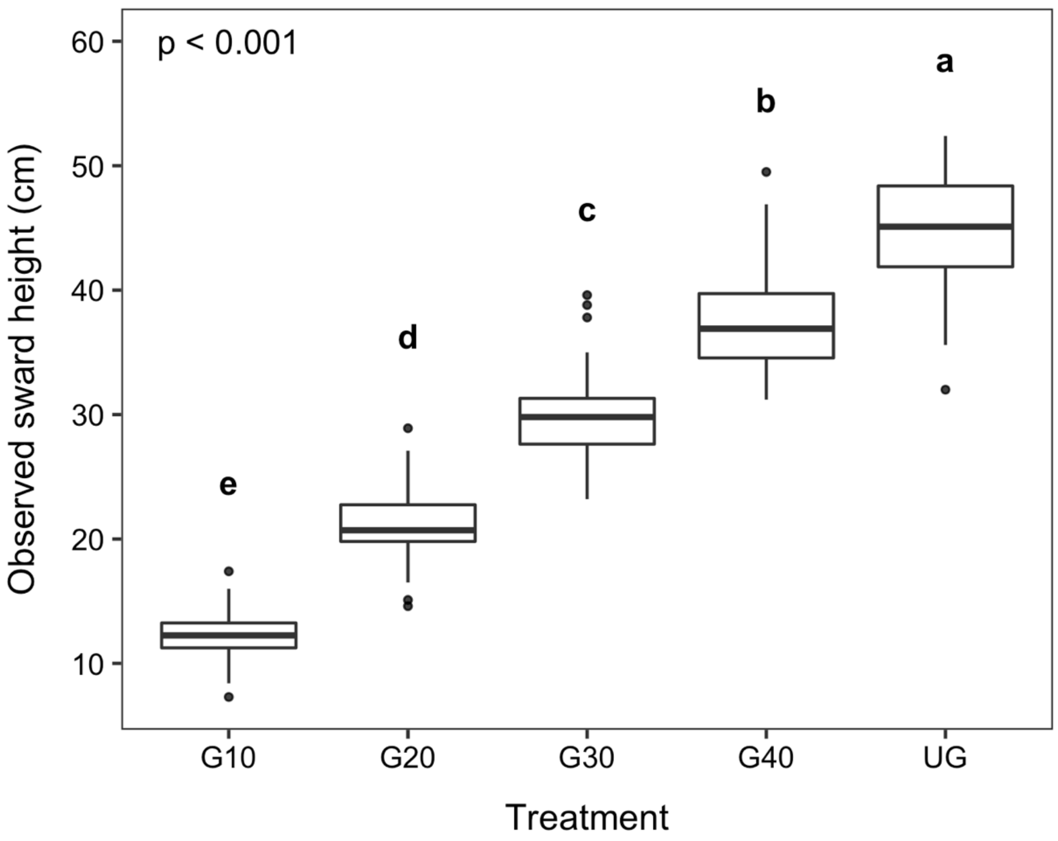

Supplementary Fig. S2. Mean sward heights (cm) for each treatment over 16 experimental years (2001-2016). G10: intense grazing (10 cm sward height); G20: moderate grazing (20 cm sward height); G30: moderate-light grazing (30 cm sward height); G40: light grazing (40 cm sward height); UG: ungrazed cover crop. Different letters indicate significant differences between means according to the Tukey test ($\alpha = 0.05$).

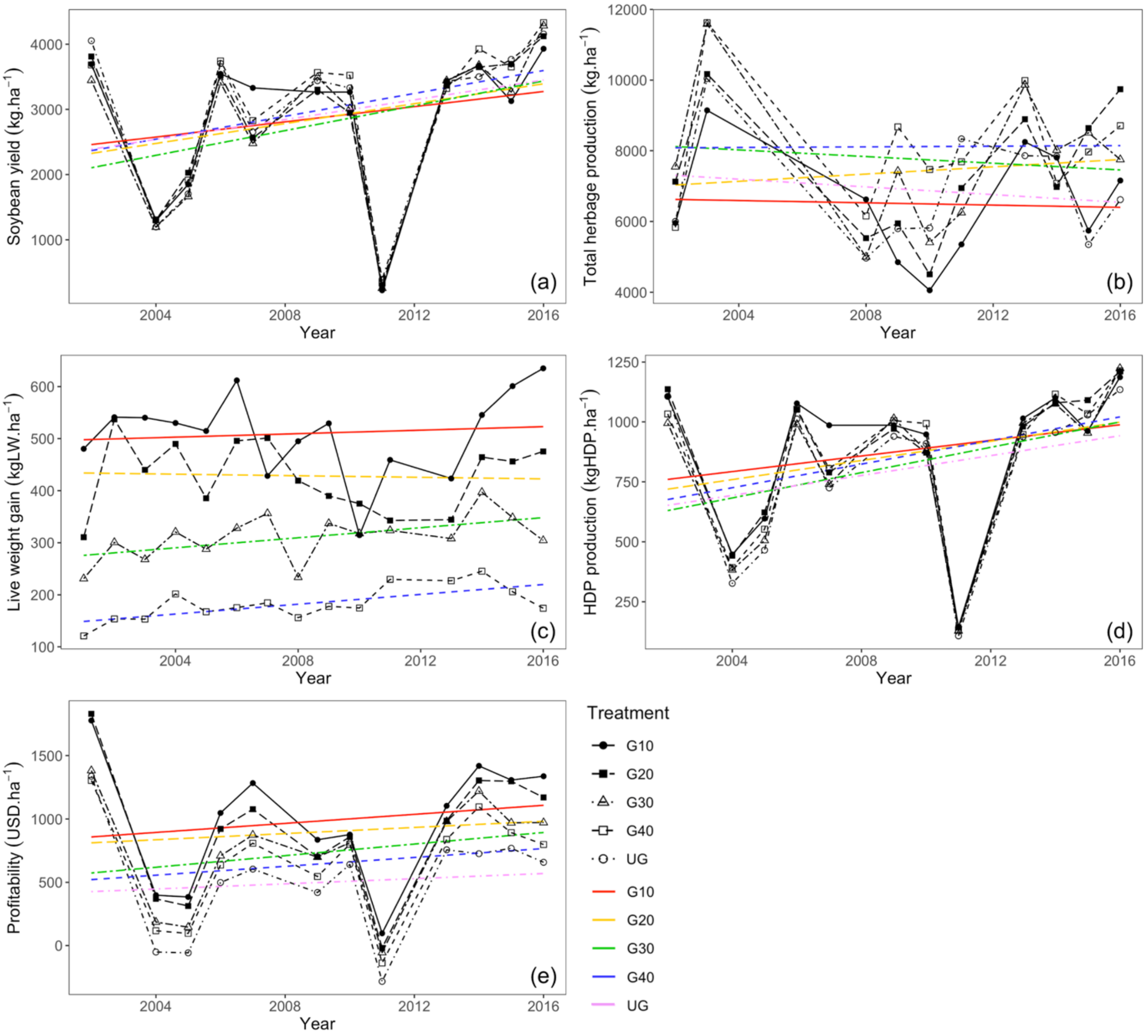

Supplementary Fig. S3. Long-term yield trajectories of (a) soybean yield (kg grain ha^{-1}), (b) total herbage production (kg dry matter ha^{-1}), (c) animal live weight (LW) gain (kg LW ha^{-1}), (d) human-digestible protein (HDP) production (kg HDP ha^{-1}) and (e) system profitability (USD ha^{-1}) from 2001 to 2016. Colored lines are the trends over the 16 years for the different treatments: G10, intense grazing (10 cm sward height); G20, moderate grazing (20 cm sward height); G30, moderate-light grazing (30 cm sward height); G40, light grazing (40 cm sward height); UG: ungrazed cover crop.

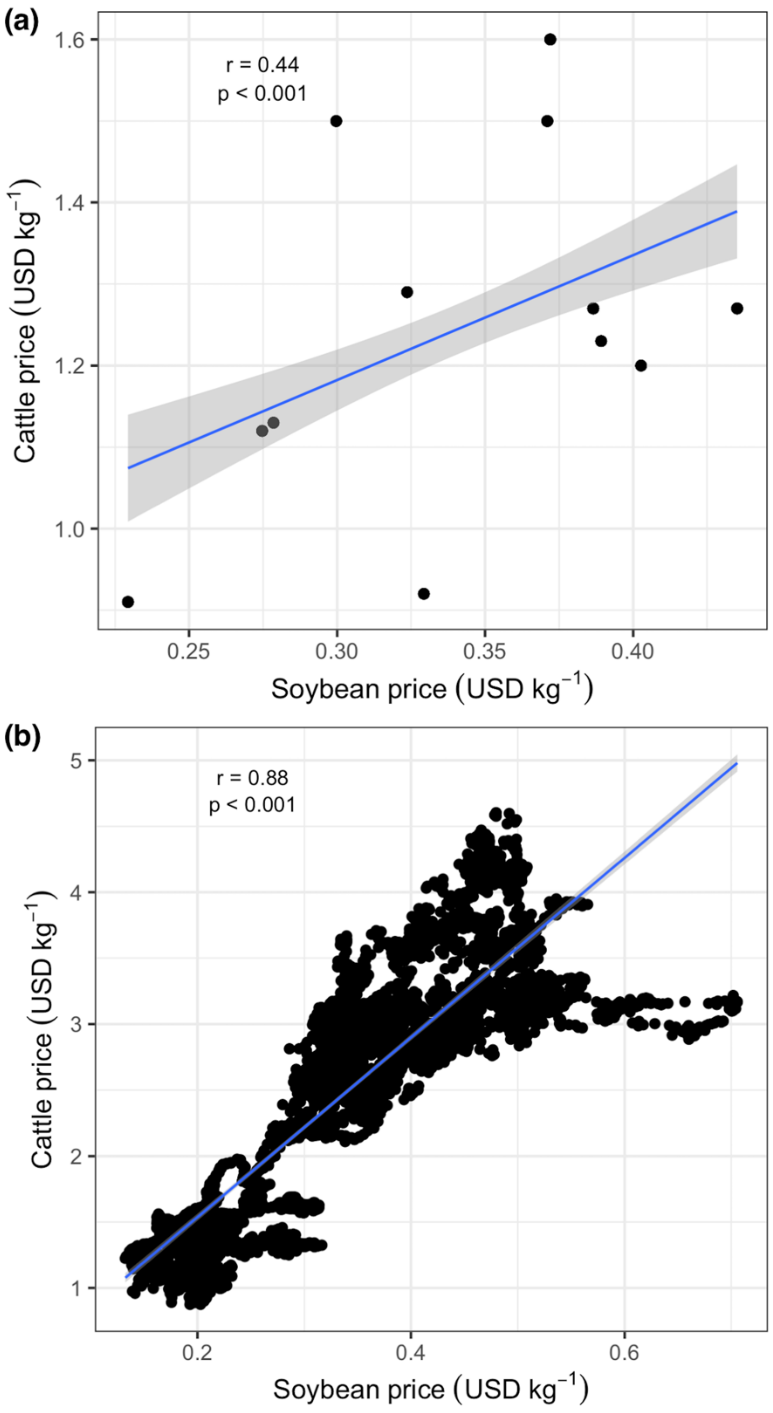

Supplementary Fig. S4. (a) Beef cattle and soybean sale prices (in November and the following April, respectively; USD kg^{-1}) used for the calculation of annual returns were positively correlated (r = 0.44, 95% confidence interval = 0.30 – 0.56, $p < 0.001$) during the experimental period. (b) Long-term daily data (1997-2020) from the Center for Advanced Studies on Applied Economics (CEPEA – University of São Paulo) confirm that cattle and soybean prices are correlated (r = 0.88, 95% confidence interval = 0.87 – 0.88, $p < 0.001$).

Supplementary Table S1. Assumed costs used for the calculation of profitability of the specialized soybean system with ungrazed cover crops in the winter (UG treatment) over the experimental period in São Miguel das Missões, Brazil.

Year	Soybean operational cost (USD ha^{-1})[a]	Cover crop operational cost (USD ha^{-1})[b]	Total cost of cropping operations (USD ha^{-1})
2002	302.36	158.05	460.41
2004	356.80	112.73	469.53
2005	375.24	92.47	467.71
2006	437.34	95.97	533.31
2007	336.31	99.99	436.30
2009	450.17	85.84	536.00
2010	341.50	86.81	428.31
2011	353.96	67.86	421.81
2013	549.10	51.90	601.00
2014	499.23	44.15	543.38
2015	561.75	36.40	598.15
2016	518.77	28.65	547.41

[a] Soybean operational cost include all the variable costs associated with crop establishment, management and post-harvest (seeds, fertilizers, weed control, harvest, storage, labor, etc.). Historic nominal values were transformed into real values using the General Market Price Index (IGP-M) from the Getúlio Vargas Foundation (FGV), Brazil, relative to November 2016.The exchange rate used for the conversion of Brazilian Reals (BRL) to U.S. Dollars (USD) was 1 BRL = 3.33 USD.

[b] Cover crop operational cost include the variable costs associated with cover crop establishment (sowing and fertilization operations). Historic nominal values were transformed into real values using the General Market Price Index (IGP-M) from the Getúlio Vargas Foundation (FGV), Brazil, relative to May 2016.The exchange rate used for the conversion of Brazilian Reals (BRL) to U.S. Dollars (USD) was 1 BRL = 3.53 USD.

Supplementary Table S2. Assumed costs used for the calculation of profitability of the integrated crop-livestock systems under different grazing intensities in the winter over the experimental period in São Miguel das Missões, Brazil.

Treatment[a]	Year	Soybean operational cost (USD ha^{-1})[b]	Livestock operational cost (USD ha^{-1})[c]	Opportunity cost of capital (USD ha^{-1})[d]	Total cost of integrated crop-livestock operations (USD ha^{-1})
G10	2002	302.36	164.64	31.85	498.85
	2004	356.80	117.42	23.13	497.35
	2005	375.24	96.32	17.93	489.49
	2006	437.34	99.97	30.25	567.56
	2007	336.31	104.16	28.53	469.00
	2009	450.17	89.42	30.93	570.52
	2010	341.50	90.43	29.47	461.40
	2011	353.96	70.68	21.56	446.20
	2013	549.10	54.06	30.16	633.32
	2014	499.23	45.99	46.60	591.82
	2015	561.75	37.92	34.34	634.01
	2016	518.77	29.84	46.54	595.15
G20	2002	302.36	162.99	24.95	490.30
	2004	356.80	116.25	17.25	490.30
	2005	375.24	95.36	13.10	483.70
	2006	437.34	98.97	19.72	556.03
	2007	336.31	103.12	24.77	464.20
	2009	450.17	88.52	18.53	557.22
	2010	341.50	89.52	22.86	453.88
	2011	353.96	69.98	17.97	441.91
	2013	549.10	53.52	23.61	626.23
	2014	499.23	45.53	31.71	576.47
	2015	561.75	37.54	23.66	622.95
	2016	518.77	29.54	31.32	579.63
G30	2002	302.36	161.34	13.68	477.38
	2004	356.80	115.08	10.96	482.84
	2005	375.24	94.40	7.90	477.54
	2006	437.34	97.97	12.85	548.16
	2007	336.31	102.08	17.58	455.97
	2009	450.17	87.63	14.44	552.24
	2010	341.50	88.62	16.04	446.16
	2011	353.96	69.27	13.84	437.07
	2013	549.10	52.98	16.49	618.57
	2014	499.23	45.07	25.19	569.49
	2015	561.75	37.16	19.00	617.91
	2016	518.77	29.24	21.78	569.79

Supplementary Table S2 (continuation). Assumed costs used for the calculation of profitability of integrated crop-livestock systems under different grazing intensities in the winter over the experimental period in São Miguel das Missões, Brazil.

Treatment[a]	Year	Soybean operational cost (USD ha^{-1})[b]	Livestock operational cost (USD ha^{-1})[c]	Opportunity cost of capital (USD ha^{-1})[d]	Total cost of integrated crop-livestock operations (USD ha^{-1})
G40	2002	302.36	159.70	8.10	470.16
	2004	356.80	113.90	7.18	477.88
	2005	375.24	93.43	5.21	473.88
	2006	437.34	96.97	6.33	540.64
	2007	336.31	101.03	8.12	445.46
	2009	450.17	86.73	7.54	544.44
	2010	341.50	87.71	8.67	437.88
	2011	353.96	68.56	8.54	431.06
	2013	549.10	52.44	12.55	614.09
	2014	499.23	44.61	14.35	558.19
	2015	561.75	36.78	11.94	610.47
	2016	518.77	28.95	13.08	560.80

[a] G10: intense grazing (10 cm sward height); G20: moderate grazing (20 cm sward height); G30: moderate-light grazing (30 cm sward height); G40: light grazing (40 cm sward height).

[b] Soybean operational cost include all the variable costs associated with crop establishment, management and post-harvest (seeds, fertilizers, weed control, harvest, storage, labor, etc.). Historic nominal values were transformed into real values using the General Market Price Index (IGP-M) from the Getúlio Vargas Foundation (FGV), Brazil, relative to November 2016.The exchange rate used for the conversion of Brazilian Reals (BRL) to U.S. Dollars (USD) was 1 BRL = 3.33 USD.

[c] Livestock operational cost include the variable costs associated with cover crop establishment (sowing and fertilization operations) and costs of animal medicines and mineral supplementation equivalent to each grazing intensity. Historic nominal values were transformed into real values using the General Market Price Index (IGP-M) from the Getúlio Vargas Foundation (FGV), Brazil, relative to May 2016.The exchange rate used for the conversion of Brazilian Reals (BRL) to U.S. Dollars (USD) was 1 BRL = 3.53 USD.

[d] Opportunity cost of capital invested in beef cattle over the different grazing intensity treatments. The opportunity cost was calculated as the product of average stocking rate, cattle price and saving account interest rate equivalent to the average number of grazing days (124 grazing days = 2% interest rate) according to the Central Bank of Brazil. Historic nominal values were transformed into real values using the General Market Price Index (IGP-M) from the Getúlio Vargas Foundation (FGV), Brazil, relative to May 2016.The exchange rate used for the conversion of Brazilian Reals (BRL) to U.S. Dollars (USD) was 1 BRL = 3.53 USD.

Supplementary Table S3. Soybean and beef cattle market prices used for the calculation of annual revenues and profitability of the specialized soybean system and the integrated crop-livestock systems under different grazing intensities by steers in the winter.

Year	Soybean price (USD kg^{-1})[a]	Beef cattle price (USD kg^{-1})[b]
2002	0.44	1.27
2004	0.33	0.92
2005	0.23	0.91
2006	0.27	1.12
2007	0.39	1.23
2009	0.28	1.13
2010	0.32	1.29
2011	0.39	1.27
2013	0.37	1.20
2014	0.40	1.50
2015	0.37	1.60
2016	0.30	1.50

[a] Soybean prices correspond to monthly averages of April in Rio Grande do Sul State, Brazil. Historic nominal prices were transformed into real values using the General Market Price Index (IGP-M) from the Getúlio Vargas Foundation (FGV), Brazil, relative to April 2017. The exchange rate used for the conversion of Brazilian Reals (BRL) to U.S. Dollars (USD) was 1 BRL = 3.13 USD.

[b] Beef cattle prices correspond to monthly averages of November in Rio Grande do Sul State, Brazil. Historic nominal prices were transformed into real values using the General Market Price Index (IGP-M) from the Getúlio Vargas Foundation (FGV), Brazil, relative to November 2016. The exchange rate used for the conversion of Brazilian Reals (BRL) to U.S. Dollars (USD) was 1 BRL = 3.33 USD.

Supplementary Table S4. Analysis of variance (ANOVA) of the model for mean soybean yield, total herbage production, animal live weight gain, human-digestible protein production and profitability.

Variable	Source	Sum of Squares	Mean Squares	Degrees of Freedom	F-value	p-value
Soybean yield	Year	30933.800	2812.160	11	313.895	< 0.001
	Treatment	43.500	10.880	4	1.215	0.375
	Year*Treatment	329.200	7.480	44	0.835	0.747
Total herbage production	Year	2.888e+08	3.209e+07	9	21.870	< 0.001
	Treatment	4.927e+07	1.232e+07	4	8.396	< 0.001
	Year*Treatment	9.4223+07	2.617e+06	36	1.784	0.015
Live weight gain	Year	2.647e+05	18907.000	14	8.007	< 0.001
	Treatment	2.3953+06	7.983e+05	3	338.099	< 0.001
	Year*Treatment	3.395e+05	8083.000	42	3.423	< 0.001
Human-digestible protein production	Year	65.802	5.982	11	448.304	< 0.001
	Treatment	0.181	0.045	4	3.386	0.065
	Year*Treatment	0.514	0.012	44	0.875	0.686
Profitability	Year	10459.0	950.82	11	238.393	< 0.001
	Treatment	1204.5	301.13	4	75.501	< 0.001
	Year*Treatment	241.3	5.48	44	1.375	0.095

Supplementary Table S5. Analysis of variance (ANOVA) of the model for yield trends of soybean yield, total herbage production, animal live weight gain, human-digestible protein production and profitability.

Variable	Source	Sum of Squares	Mean Squares	Degrees of Freedom	F-value	p-value
Soybean yield	Year	1.383e+22	1.383e+22	1	24.042	< 0.001
	Treatment	1.250e+21	3.125e+20	4	0.543	0.704
	Year*Treatment	1.250e+21	3.125e+20	4	0.543	0.704
Total herbage production	Year	4.281e+05	4.281e+05	1	0.106	0.745
	Treatment	4.111e+06	1.028e+06	4	0.254	0.907
	Year*Treatment	4.125e+06	1.031e+06	4	0.255	0.906
Live weight gain	Year	26904.000	26903.900	1	5.466	0.021
	Treatment	22017.000	7339.100	3	1.491	0.219
	Year*Treatment	21169.000	7056.300	3	1.434	0.235
Human-digestible protein production	Year	7.539e+18	7.539e+18	1	24.090	< 0.001
	Treatment	6.568e+17	1.642e+17	4	0.525	0.718
	Year*Treatment	6.574e+17	1.643e+17	4	0.525	0.717
Profitability	Year	780929	780929	1	3.501	0.063
	Treatment	105636	26409	4	0.118	0.976
	Year*Treatment	106994	26748	4	0.120	0.975
